# The interleukin-1 beta 511C>T polymorphism is associated with susceptibility to juvenile idiopathic arthritis in a Turkish population

**DOI:** 10.1590/1806-9282.20251421

**Published:** 2026-05-01

**Authors:** Seyda Dogantan, Yasemin Oyaci, Figen Cakmak, Sevde Hasanoğlu Sayin, Emel Hatun Aytaç Kaplan, Sacide Pehlivan

**Affiliations:** 1 Başakşehir Çam ve Sakura City Hospital, Department of Pediatric Rheumatology – Istanbul, Türkiye.; 2 Istanbul University, Faculty of Medicine, Department of Medical Biology – Istanbul, Türkiye.; 3 Başakşehir Çam ve Sakura City Hospital, Department of Pediatric Endocrinology – Istanbul, Türkiye.

**Keywords:** Juvenile idiopathic arthritis, Genetic predisposition, Interleukin-1 beta, Single nucleotide polymorphism, Turkey, Case-control studies

## Abstract

**BACKGROUND:**

Juvenile idiopathic arthritis is the most common chronic rheumatic disease of childhood, with a significant genetic component. Polymorphisms in proinflammatory cytokine genes are potential contributors to disease susceptibility. The aim of this study was to investigate the association between *tumor necrosis factor-alpha (TNF-α*)-308G>A (rs1800629), *interleukin-1 beta (IL-1β)*-511C>T (rs16944), and *interleukin-6 (IL-6)*-174G>C (rs1800795) variants and juvenile idiopathic arthritis in a Turkish cohort.

**METHODS:**

A case–control study was conducted involving 57 juvenile idiopathic arthritis patients and 50 age- and sex-matched healthy controls. Genotyping was performed using polymerase chain reaction–restriction fragment length polymorphism. Genotype and allele distributions were compared between groups, and associations with clinical parameters (erythrocyte sedimentation rate, C-reactive protein, disease subtype) were assessed.

**RESULTS:**

A significant association was found between the *IL-1β*-511C>T polymorphism and juvenile idiopathic arthritis. The CC genotype was significantly more frequent in patients compared to controls (24.6 vs. 2.0%; p=0.004), and the C allele demonstrated a trend toward association (p=0.051). In contrast, no significant differences were observed in the genotype or allele frequencies of the *TNF-α*-308G>A or *IL-6*-174G>C variants between patients and controls. Furthermore, none of the studied polymorphisms were associated with erythrocyte sedimentation rate, C-reactive protein levels, or specific disease subtypes.

**CONCLUSION:**

This study identifies the *IL-1β*-511C>T polymorphism as a significant risk factor for juvenile idiopathic arthritis in the Turkish population, underscoring the role of the IL-1 pathway in disease pathogenesis. These findings contribute to the understanding of the ethnic-specific genetic architecture of juvenile idiopathic arthritis and suggest *IL-1β* as a potential candidate for further functional and pharmacogenetic studies.

## INTRODUCTION

Juvenile idiopathic arthritis (JIA), a complex autoimmune disease, is characterized by persistent chronic arthritis. This condition, which includes all types of chronic arthritis, usually lasts longer than 6 weeks and develops before the age of 16^1^. Single-nucleotide polymorphisms (SNPs) in both coding and non-coding areas of the genome have been the subject of disease-association research on rheumatoid arthritis (RA) and JIA. This has allowed for the identification of some SNPs that are currently thought to be markers of disease severity and response to treatment^2^.

Tumor necrosis factor-α (TNF-α) promotes inflammation and immune activation. The −308G>A (rs1800629) variant enhances TNF-α expression, increasing protein levels and disease severity in JIA, especially systemic and RF-positive polyarticular types^1,3,4^.

The interleukin-1 (IL-1) cluster has become a strong non-MHC (major histocompatibility complex) susceptibility locus and has been linked to the mediation of acute and severe inflammatory illnesses^5^. The *IL-1α*, *IL-1β*, and *IL-1RN* genes are the archetypal members of the IL-1 family gene cluster. The *IL-1B* gene is highly polymorphic, and base transitions between C and T at positions *-511* (C/T; dbSNP: rs16944) have been related to increased IL-1β secretion^6^.

Interleukin-6 (IL-6), a key cytokine in innate and adaptive immunity, influences Th17 cell differentiation in JIA. The −174G>C (rs1800795) promoter variant affects IL-6 expression. The IL-6 −174G>C C allele has been associated with increased RA risk in Egyptians^7–10^.

Therefore, we aimed to investigate whether variants in *TNF-α* −308G>A, *IL-1*β −511C>T, and *IL-6* −174G>C are associated with JIA in Turkey. We also evaluated the relationship between clinical findings and genotype distribution.

## METHODS

### Study population

In this study, 57 JIA patients (31 females and 26 males; mean age: 14) were recruited between July 2024 and September 2024 from the Rheumatology Clinic of the Pediatrics Department, Başakşehir Çam and Sakura Hospital, Istanbul, Turkey. In order to illustrate the diagnosis of JIA, we employed the International League of Associations for Rheumatology (ILAR) categorization criteria^10^. Patients with other autoimmune diseases, cancer, or recent infections were excluded. JIA patients underwent a detailed clinical evaluation. Demographic and clinical data were recorded. In total, 50 (29 females and 21 males; mean age: 14) age-, sex-, and ethnicity-similar healthy individuals without chronic illness served as the control group. All participants and their parents submitted informed written consent before enrolling in the study, based on the ethical guidelines of the 2008 Declaration of Helsinki, and the local Başakşehir Çam and Sakura Hospital ethical committee approved the investigation (Approval Number: E-96317027-514.10-245113237).

### Genotyping analysis

To analyze these variations, roughly 5 mL of peripheral blood was drawn. Using the QIAamp DNA Blood Mini Kit (Qiagen GmbH, Hilden, Germany), DNA is extracted from leukocytes. *TNF-α* −308G>A, *IL-1β* −511C>T, and *IL-6* −174G>C variants were genotyped by polymerase chain reaction (PCR) and restriction fragment length polymorphism (RFLP) methods as described previously^11,12^.

### Statistical analysis

Statistical analyses were performed using OpenEpi v3.01 and SPSS v20. Data were presented as frequency [n (%)]. χ² test, and Fisher's exact test were also used. A p<0.05 was considered statistically significant.

## RESULTS

In the present study, a total of 107 subjects, including 57 JIA patients and 50 healthy subjects, were genotyped for the variants.

Allelic and genotypic distributions of the *TNF-α* −308G>A, *IL-1β* −511C>T, and *IL-6* −174G>C variants in patients and controls are shown in Table 1. There were no statistically significant differences between patients and healthy controls in the allele and genotype frequencies of the *TNF-α*-308G>A and *IL-6* −174G>C variants (p>0.05).

**Table 1:** Genotype and allele frequencies of *TNF- α* −308G>A, *IL-1 β* −511C>T, and *IL-6* - 174G>C variants in groups.

		Patients n: 57 (%)	Controls n: 50 (%)	p-value
TNF-α −308G>A				
Genotypes				
	GG	10 (17.5)	10 (20)	0.707*
	AG	0 (0)	1 (2)	
	AA	47 (82.5)	39 (78)	
Alleles				
	G	20 (17.5)	21 (21)	0.522*
	A	94 (82.5)	79 (79)	
IL-1ß −511C>T				
Genotypes				
	TT	9 (15.8)	10 (20.0)	**0.004** *
	CT	34 (59.6)	39 (78.0)	
	CC	14 (24.6)	1 (2.0)	
Alleles				
	T	52 (45.6)	59 (59.0)	**0.051** *
	C	62 (54.4)	41 (41.0)	
IL-6 −174G>C				
Genotypes				
	CC	1 (1.8)	1 (2)	0.990*
	GC	20 (35.1)	18 (36)	
	GG	36 (63.1)	31 (62)	
Alleles				
	C	22 (19.3)	20.0 (20)	0.897*
	G	92 (80.7)	80.0 (80)	

* Chi-square test/Fisher's exact test.

The results that are statistically significant are shown in boldface.

The distribution of genotype and allele frequencies of the *IL-1β* −511C>T variant was statistically different between patients and the control group. The *IL-1β* −511C>T CC genotype was higher in patients compared to controls, while the TT and CT genotypes were more common in the control group than in patients (p=0.004). The *IL-1β* −511C>T variant C allele was higher in patients than in controls (p=0.051).

Then, we compared genotype distributions in the patient group according to erythrocyte sedimentation rate (ESR), C-reactive protein (CRP), and disease types (Tables 2 and 3). There were 43 patients with ESR levels below 20 and 14 patients with ESR values above 20. *TNF-α* −308G>A, *IL-1β* −511C>T, and *IL-6* −174G>C genotype distributions were not statistically significant in patients with ESR levels below 20 and above 20 (p=1.000, p=0.095, and p=1.000, respectively). There were 42 patients with CRP levels <5 and 15 patients with CRP values ≥5. The genotype distribution of these variants did not differ according to CRP status (p=1.000, p=1.000, and p=1.000, respectively).

**Table 2:** Genotype distributions of variants according to erythrocyte sedimentation rate and C-reactive protein status.

	ESR<20	ESR≥20	p-value
	n: 43 (%)	n: 14 (%)	
TNF-α −308G>A			
GG	8 (18.6)	2 (14.3)	1.000*
AG/AA	35 (81.4)	12 (85.7)	
IL-1ß −511C>T			
TT	9 (20.9)	0 (0)	0.095*
CT/CC	34 (79.1)	14 (100)	
IL-6 −174G>C			
CC	1 (2.3)	0 (0)	1.000*
GC/GG	42 (97.7)	14 (100)	
	**CRP<5**	**CRP≥5**	**p-value**
	**n=42 (%)**	**n=15 (%)**	
TNF-α −308G>A			
GG	8 (19.0)	2 (13.3)	1.000*
AG/AA	34 (81.0)	13 (86.7)	
IL-1ß −511C>T			
TT	7 (16.7)	2 (13.3)	1.000*
CT/CC	35 (83.3)	13 (86.7)	
IL-6 −174G>C			
CC	1 (2.4)	0 (0)	1.000*
GC/GG	41 (97.6)	15 (100)	

* Chi-square test/Fisher's exact test. ESR: erythrocyte sedimentation rate; CRP: C-reactive protein.

**Table 3:** Genotype distributions of variants according to disease types.

	Oligoarticular JIA	Enthesit-associated JIA	Polyarticular JIA	Systemic JIA	Psoriatic JIA	p-value
	n: 20 (%)	n: 19 (%)	n: 9 (%)	n: 5 (%)	n: 4 (%)	
TNF-α −308G>A						
GG	3 (15)	4 (21.1)	1 (11.1)	0 (0)	2 (50)	0.410*
AG/AA	17 (85)	15 (78.9)	8 (88.9)	5 (100)	2 (50)	
IL-1ß −511C>T						
TT	7 (35.0)	1 (5.3)	1 (11.1)	0 (0)	0 (0)	0.105*
CT/CC	13 (65.0)	18 (94.7)	8 (88.9)	5 (100)	4 (100)	
IL-6 −174G>C						
CC	0 (0)	0 (0)	0 (0)	1 (20)	0 (0)	0.158*
GC/GG	20 (100)	19 (100)	9 (100)	4 (80)	4 (100)	

* Chi-square test/Fisher's exact test. JIA: juvenile idiopathic arthritis.

## DISCUSSION

JIA encompasses chronic pediatric arthritis, affecting joints and extra-articular organs. The ILAR classifies JIA subtypes based on joint involvement, systemic features, and rheumatoid factor (RF) status^13^. Each of the several subtypes of JIA has a distinct genetic predisposition and level of arthritis severity. Systemic JIA (sJIA), polyarticular (RF-positive or RF-negative), oligoarticular (permanent or extended), psoriatic, and enthesitis-related arthritis are these subtypes^1^. JIA subtypes share chronic joint inflammation despite clinical differences. Genetic and environmental factors influence susceptibility. Dysregulation of both innate and adaptive immunity leads to inflammation, with monocytes, macrophages, fibroblasts, and T cells releasing proinflammatory mediators that contribute to disease pathogenesis.

TNF-α is a crucial cytokine in the pathophysiology of JIA, and increased TNF-α in JIA may cause bone loss, cartilage loss, and tissue damage^14^. It can trigger the development of proinflammatory cytokines such as IL-1β, IL-6, and IL-8, resulting in a prolonged inflammatory response^15^. However, a study reported that blood TNF-α concentration in children with oligoarticular JIA has no diagnostic value in monitoring the severity of the disease or effectiveness of treatment^16^. A statistically significant difference in the frequency of the *TNF-α* −308 GG genotype was reported in both co-dominant and dominant models between psoriatic arthritis (PsA) patients and healthy controls in the Kuwaiti population^17^. Gazzar et al. studied the Egyptian population and found that the serum TNF-α levels were significantly higher in patients. They discovered that the *TNF-α* −308G>A GG genotype was more likely to be associated with the systemic onset subtype of the condition, as opposed to the oligoarticular and polyarticular subtypes^14^. Similarly, in the Saudi population, the *TNF-α* −308 G allele and GG genotype were found to be higher in RA patients than in the control group1^8^. Research has demonstrated that JIA patients with the *TNF-α* −308 GA/AA genotypes had a poorer prognosis and a reduced responsiveness to anti-TNF-α medications^1^. In this study, the *TNF-α* −308G>A genotype and allele distribution did not differ between patients and controls. Similarly, *TNF-α* −308G>A genotype distribution was not different in patients according to ESR level, CRP level, and disease types.

The *IL-1β* gene codes for IL-1β, an efficient proinflammatory cytokine that plays a key role in both bone resorption and the breakdown of joint structures^19^. It is believed that IL-1β is expressed by activated macrophages in sensory joints and may trigger the activation of T helper 17 cells as well as the enrollment of other proinflammatory immunological indices like IL-6 and TNF-α^20^. It was detected that the *IL-1β* gene, −511CC genotype, was observed in RA patients in comparison to controls^21^. Ziaee et al. observed no significant difference between the JIA patients and controls for *IL-1β* −511 C>T in the Iranian population^22^. In this study, we found that the *IL-1ß* −511C>T CC genotype was higher in the patient group than in the control group. One possible explanation is that the variation C allele leads to abnormal immunological reactions and altered expression of the *IL-1β* gene, which predisposes an individual to JIA vulnerability. Furthermore, the placement of this SNP in the promoter region may have an impact on the transcription factor binding site's structure, which in turn may have an impact on the generation of *IL-1β*. The p-value obtained for the genotype distribution of the *IL-1β* −511C>T variant is significant, but the p-value at the allele level is borderline. This suggests that the effect of this variant may arise through a specific genetic model rather than a simple difference in allele frequency. This situation indicates that the C allele may be associated with a recessive effect model and that disease risk is more pronounced only in homozygous genotypes. However, *IL-1β* −511 C>T genotype distribution did not differ according to clinical features, including ESR level, CRP level, and disease types.

Reduced disease activity and slower radiographic development of RA have been observed when IL-6 activity is blocked using the soluble anti-IL-6 molecule tocilizumab^23^. It was observed that the "G" allele in *IL-6* −174G>C increases transcriptional activity, which raises IL-6 levels in RA in both blood and synovial fluid^17^. Al-Awadhi et al. showed an association between the *IL-6* −174 C allele and PsA in the Kuwaiti population^17^. A meta-analysis conducted by Lee et al. found an association between the *IL-6* −174 G allele and RA in the European population^24^. Ziaee et al. demonstrated that the *IL-6* −174 G allele was higher in patients with JIA compared to controls in the Iranian population^22^. In the present study, we found no significant difference between the groups of patients and controls for *IL-6* −174G>C. ESR level, CRP level, and disease types were also not found to be associated with *IL-6* −174G>C genotype distribution.

Recent metabolomic studies highlight the heterogeneous biological nature of JIA and the presence of distinct molecular profiles even in treated patients, revealing the genetic and biological diversity of the disease^25^. In this context, our study demonstrates that the *IL-1β*-511C>T polymorphism may be an important genetic marker for JIA specific to the Turkish population. By supporting the critical role of the IL-1 pathway in pathogenesis, it provides a valuable foundation for future functional and pharmacogenetic studies.

This study has several limitations. The first limitation is that it evaluated only three variants of the cytokine genes. Other variants of these genes may also affect the development of JIA. In addition, the gene–environment and gene–gene interactions were not studied for these variants since there is no original information. Another limitation of the study is the relatively small sample size. The failure to identify any relationship with clinical parameters may also be related to this situation. This is because dividing the sample into subgroups further reduces the number of individuals in each group, decreases statistical power, and may prevent the emergence of significant differences. The final limitation of this study is that the blood level of these cytokines was not assessed. However, the fact that this single-center study reflects data from a certain region increases the value of the study.

## CONCLUSION

In conclusion, the current case–control study suggests that the promoter variant of the *IL-1ß* −511C>T gene plays an important role in the development of JIA in the Turkish population. On the other hand, there is no significant relationship between *TNF-α* 308G>A or *IL-6* −174G>C and JIA predisposition. Due to the relationship between *IL-1ß* −511C>T and JIA, it would be useful to examine this gene further.

## Data Availability

The datasets generated and/or analyzed during the current study are available from the corresponding author upon reasonable request.
